# Genomic and Functional Characterization of Acetolactate Synthase (ALS) Genes in Stress Adaptation of the Noxious Weed *Amaranthus palmeri*

**DOI:** 10.3390/plants14193088

**Published:** 2025-10-07

**Authors:** Jiao Ren, Mengyuan Song, Daniel Bimpong, Fulian Wang, Wang Chen, Dongfang Ma, Linfeng Du

**Affiliations:** 1 MARA Key Laboratory of Sustainable Crop Production in the Middle Reaches of the Yangtze River (Co-construction by Ministry and Province)/Hubei Key Laboratory of Waterlogging Disaster and Agricultural Use of Wetland, College of Agriculture, Yangtze University, Jingzhou 432000, China; renjiaogl@163.com (J.R.); ssmmyy0213@163.com (M.S.); bimpong81@gmail.com (D.B.); wangfl_hb@163.com (F.W.); chenwangchw@163.com (W.C.); 2 Key Laboratory of Integrated Pest Management of Crops in Central China, Ministry of Agriculture/Hubei Key Laboratory of Crop Diseases, Insect Pests and Weeds Control, Institute of Plant Protection and Soil Science, Hubei Academy of Agricultural Sciences, Wuhan 430064, China; 3 Hubei Provincial National Forest Farm Workstation, Wuhan 430070, China

**Keywords:** weed, abiotic stress, herbicide response, gene expression, chlorophyll

## Abstract

Acetolactate synthase (ALS) is an important enzyme in plant branched-chain amino acid biosynthesis and the target of several major herbicide classes. Despite its agronomic importance, the role of ALS genes in stress adaptation in the invasive weed *Amaranthus palmeri* remains unstudied. In this study, four *ApALS* genes with high motif conservation were identified and analyzed in *A. palmeri*. Phylogenetic analysis classified ApALS and other plant ALS proteins into two distinct clades, and the ApALS proteins were predicted to localize to the chloroplast. Gene expression analysis demonstrated that *ApALS* genes are responsive to multiple stresses, including salt, heat, osmotic stress, glufosinate ammonium, and the ALS-inhibiting herbicide imazethapyr, suggesting roles in both early and late stress responses. Herbicide response analysis using an *Arabidopsis thaliana* ALS mutant (*AT3G48560*) revealed enhanced imazethapyr resistance, associated with higher chlorophyll retention. Furthermore, high sequence homology between *AT3G48560* and *ApALS1* suggests a conserved role in protecting photosynthetic function during herbicide stress. This study provides the first comprehensive analysis of the ALS gene family in *A. palmeri* and offers important insights into its contribution to stress resilience. These findings establish a vital foundation for developing novel strategies to control this pervasive agricultural weed and present potential genetic targets for engineering herbicide tolerance in crops.

## 1. Introduction

The genus Amaranthus (Amaranthaceae) comprises approximately 75 species globally. *Amaranthus palmeri* S. Watson (Palmer amaranth) is an annual, invasive plant native to a region spanning northwestern Mexico and the southwestern United States, including southern California, New Mexico, and Texas [1]. This weed poses a significant threat to agriculture due to its prolonged emergence period, which coincides with the growing seasons of major row crops, and its rapid growth rate, which enables intense competition for resources. *Amaranthus palmeri* can accumulate substantial aboveground biomass, severely inhibiting crop development. Consequently, the season-long interference of *A. palmeri* has been shown to cause significant yield losses in corn (*Zea mays* L.), cotton (*Gossypium hirsutum* L.), and soybean (*Glycine max* (L.) [2,3].

Over the past two decades, reports of both global invasion and herbicide resistance in *A. palmeri* have increased markedly. The species began spreading from its native range via agricultural production and grain transport in the early 20th century, subsequently impacting agriculture in invaded regions [4]. A significant milestone occurred a decade after the widespread adoption of transgenic crops, with the first documentation of glyphosate-resistant *A. palmeri* in Georgia and North Carolina, United States [5]. Following this initial report, resistant populations were rapidly identified across increasingly widespread areas of the USA. Many USA populations now exhibit multiple herbicide resistance (MHR), encompassing mechanisms of action that include ALS inhibitors, 5-enolpyruvylshikimate-3-phosphate synthase (EPSPS) inhibitors, 4-hydroxyphenylpyruvate dioxygenase (HPPD) inhibitors, long-chain fatty acid inhibitors, microtubule assembly inhibitors, photosystem-II inhibitors, protoporphyrinogen oxidase (PPO) inhibitors, and synthetic auxins [6]. In China, *A. palmeri* was first recorded in 1985 in Beijing’s Fengtai District [7]. Populations with high-level resistance to herbicides such as imazethapyr are already established domestically. The primary resistance mechanism identified is target-site mutation, specifically mutations in the acetolactate synthase (ALS) gene [8]. However, comprehensive genomic analyses of the herbicide-resistant ALS gene family in *A. palmeri* have not yet been studied.

ALS is a nuclear-encoded flavoprotein that is post-translationally targeted to the chloroplast. It functions as the rate-limiting enzyme in the biosynthetic pathway for the branched-chain amino acids (BCAAs) valine, leucine, and isoleucine. ALS catalyzes one of two initial condensation reactions: the joining of two pyruvate molecules to form acetolactate (a precursor to valine and leucine) or the condensation of pyruvate with 2-ketobutyrate to yield 2-acetohydroxybutyrate (a precursor to isoleucine) [9,10,11]. ALS is the target site for several major classes of selective herbicides, known collectively as ALS inhibitors. These herbicides function by binding to the enzyme’s active site within the chloroplast, hindering substrate access and inhibiting catalytic activity [12,13]. This inhibition occurs in the chloroplast stroma (cytoplasmic matrix of the organelle) and results in a rapid depletion of BCAAs. The ensuing deficiency disrupts essential cellular processes, primarily protein synthesis and cell division, ultimately leading to plant death in susceptible species [14,15]. Based on their distinct chemical structures, ALS-inhibiting herbicides are classified into seven principal categories: imidazolinones, sulfonylureas, triazolopyrimidines-types 1, triazolopyrimidines-type 2, triazolinones, pyrimidinylbenzoates, and sulfonanilides [16].

These ALS-inhibiting herbicides are widely used in global agriculture owing to their high efficacy at low application rates and their favorable toxicological profile for mammals [17]. Their mechanism of action involves binding to the ALS enzyme, which sterically hinders substrate access to the catalytic site. This inhibition halts branched-chain amino acid production, leading to plant growth arrest and eventual mortality [18]. Given that ALS inhibitors constitute over one-sixth of all registered herbicides globally, the ALS gene has become a primary target for developing herbicide-resistant crops [19]. Target-site resistance in weeds was first documented in 1987, when prickly lettuce (*Lactuca serriola* L.) was found to possess an ALS gene mutation conferring resistance after selection pressure from an ALS-inhibitor herbicide [20]. Herbicides that target specific biochemical pathways, such as ALS inhibitors, exhibit high molecular specificity. This precision, while effective for weed control, also contributes to a relatively elevated baseline frequency of resistance alleles within native weed populations. For instance, in *A. thaliana*, the initial frequency of such alleles was estimated at 3.2 × 10^−5^ [21], indicating that resistant individuals may already exist prior to herbicide application. This pre-existing genetic variation accelerates the selection process under herbicide pressure, making these compounds particularly prone to resistance evolution. As a result, this herbicide group currently accounts for the highest number of resistance cases globally, with 176 unique instances and a total of 746 reported cases [22]. Therefore, investigating the function and properties of the ALS gene in *A. palmeri* is essential for understanding the molecular basis of its significant herbicide resistance.

Despite extensive documentation of *A. palmeri* invasiveness and its evolving resistance to multiple herbicide modes of action, the molecular mechanisms underlying this resistance remain poorly understood. In particular, the functional roles of *ApALS* genes in stress regulation have not yet been investigated. This lack of comprehensive insight into *ApALS* gene diversity and function constrains our ability to fully understand the mechanisms driving resistance and impedes the development of targeted management strategies. To address this gap, this study will identify and characterize *ALS* genes in *A. palmeri*. Bioinformatics approaches were employed to identify *ApALS* gene family members and analyze the physicochemical properties of their encoded proteins. The expression patterns of *ApALS* genes were evaluated under diverse abiotic stresses, including salt (NaCl), heat, PEG6000-induced osmotic stress (simulating drought), and herbicide treatments (glufosinate ammonium and imazethapyr). Furthermore, the subcellular localization of ApALS proteins was characterized, and functional complementation assays were performed by expressing *A. thaliana ALS* mutants exposed to imazethapyr stress. The findings from this research establish a key foundation for future investigations into *ApALS* genes, which could provide strategies for managing *A. palmeri* expansion. Additionally, this knowledge may contribute to the development of stress-resistant crop varieties through the molecular breeding of economically important species.

## 2. Results

### 2.1. Identification, Chromosome Mapping, and Phylogenetic Analyses of ApALS Proteins

**Figure 1:**
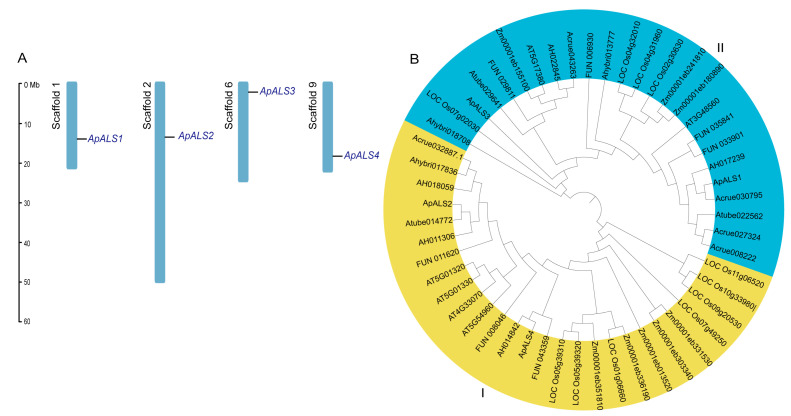
Chromosome mapping and phylogenetic analysis of ApALS proteins. Chromosome distribution of ApALSs across the scaffolds of *A. palmeri*. The scaffold names are indicated in black, while the ApALSs are highlighted in blue. The length of the scaffolds is represented in megabases (Mb) (**A**). Phylogenetic tree of ALS proteins from *A. palmeri*, *A. cruentus*, *A. tuberculatus*, *A. hybridus*, *A. hypochondriacus*, *A. thaliana*, *O. sativa*, *P. amilis*, and *Z. mays*. The nodes in the tree were evaluated using bootstrap analysis with 1000 replicates (**B**).

Initial identification using dual BLAST approaches using TBtools v2.310 found six candidate ApALS protein sequences. Subsequent validation screening with the NCBI CDD and SMART databases confirmed that four of these ApALSs encoded the definitive TPP_enzyme_N, TPP_enzyme_M, TPP_enzyme_C, and PLN domains characteristic of functional ALS proteins. To determine their genomic distribution, the four *ApALSs* were mapped to their respective *A. palmeri* genome scaffolds based on coordinates provided in the GFF annotation file. Each gene was located on a separate scaffold (Figure 1A).

To investigate evolutionary relationships within the ALS family, a phylogenetic tree was constructed using a dataset of 52 ALS protein sequences, including representatives from multiple species: 4 ApALSs, 5 *A. cruentus* (AcALSs), 3 *A. tuberculatus* (AtuALSs), 3 *A. hybridus* (AhALSs), 5 *A. hypochondriacus* (AhyALSs), 6 *A. thaliana* (AtALSs), 11 *Oryza sativa* (OsALSs), 7 *Portulaca amilis* (PaALSs), and 8 *Zea mays* (ZmALSs). The analysis clustered the proteins into two well-defined phylogenetic subgroups (Figure 1B). Subgroup I contained 27 proteins, and Subgroup II contained 25. The ApALS proteins were distributed across both subgroups. Notably, close evolutionary relationships were observed, as ApALS2, ApALS4, and ApALS1 clustered on terminal branches with Atube014772, AH014842, and Acrue030795, respectively.

### 2.2. Protein Features of ApALS Proteins

The physicochemical properties of the identified ApALS amino acid sequences range from 354 to 669 aa, with an average of 554.75 aa. Corresponding molecular weights varied considerably from 37.74 to 72.66 kDa, averaging 60.07 kDa. The theoretical isoelectric points (pI) ranged from 5.67 to 6.88 (average pI = 6.26), classifying these proteins as neutral. The instability indices, averaging 36.66 (range: 30.42–42.75), indicated generally stable proteins. The grand average of hydropathicity (GRAVY) values ranged from -0.138 to 0.06, with a mean of -0.035; the majority of ApALS proteins were hydrophilic (GRAVY < 0). No signal peptides were predicted; however, all *ApALSs* were predicted to possess chloroplast transit peptides (Supplementary File S2).

**Figure 2:**
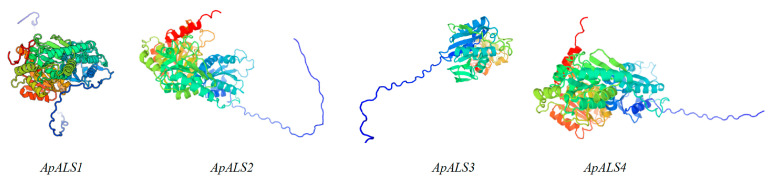
Predicted 3-dimensional models of ApALS proteins using SWISS-MODEL.

Analysis of secondary structures revealed four common elements: alpha helices, extended strands, beta turns, and random coils. Alpha helices (30.64–39.55%) and random coils (35.59–47.09%) constituted the predominant structures, while beta turns (5.16–6.78%) and extended strands (15.32–18.08%) were less abundant (Supplementary File S3). Homology modeling performed using the SWISS-MODEL server indicated that all ApALS proteins adopt complex tertiary structures characteristic (Figure 2).

### 2.3. Gene Structure, Conserved Motifs, Cis-Acting Elements, and miRNA Targets of ApALSs

**Figure 3:**
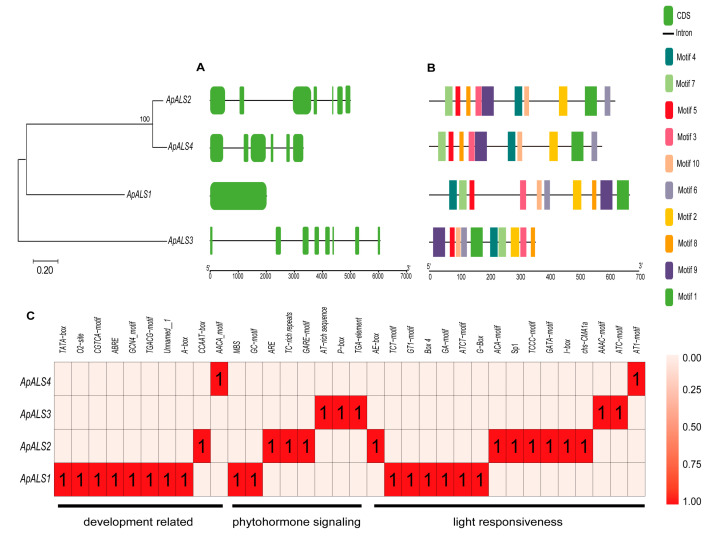
Gene structure, conserved motifs, and *cis*-acting regulatory elements of *ApALS* genes. Structural characteristics of *ApALS*s (**A**). Conserved motifs of ApALS proteins (**B**). *Cis*-acting regulatory elements within 2 kb upstream of the *ApALS*s promoter region (**C**).

**Figure 4:**
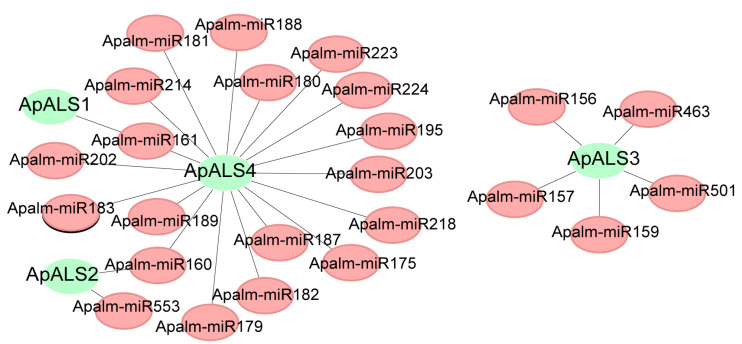
MiRNA-*ApALS* target interaction network, with *ApALS* represented in green and miRNAs in red.

Analysis of gene structure revealed considerable heterogeneity in exon–intron organization among the *ApALS* family members. *ApALS* (2, 3, and 4) contains 6–8 exons and 5–7 introns, whereas *ApALS1* harbors a single exon structure. Notably, no untranslated regions (UTRs) were identified in the structure of the *ApALSs* (Figure 3A). Conserved motif analysis identified 10 putative motifs, ranging from 15 to 41 amino acids in length, with each motif appearing once per sequence. All ten motifs were present in every ApALS protein (Figure 3B; Supplementary File S4). Examination of the 2.0 kb promoter regions upstream of the *ApALS* genes identified numerous *cis*-acting regulatory elements. These were predominantly associated with three categories: light responsiveness, phytohormone signaling, and stress adaptation. Light-responsive elements were ubiquitous, though their abundance varied significantly, with *ApALS1* containing the highest number and *ApALS4* the lowest (Figure 3C). Furthermore, the promoters harbored multiple elements responsive to phytohormones, including gibberellin (GA), salicylic acid (SA), and abscisic acid (ABA). To investigate the potential regulatory mechanisms of *ApALS* genes, their interactions with microRNAs (miRNAs) were analyzed. A total of 24 miRNAs were predicted to target the four *ApALS* genes (Figure 4; Supplementary File S5). The number of miRNAs targeting each gene varied widely, from one miRNA for *ApALS1* to 18 for *ApALS4*.

### 2.4. ApALS Expression Analysis Under Different Treatments

**Figure 5:**
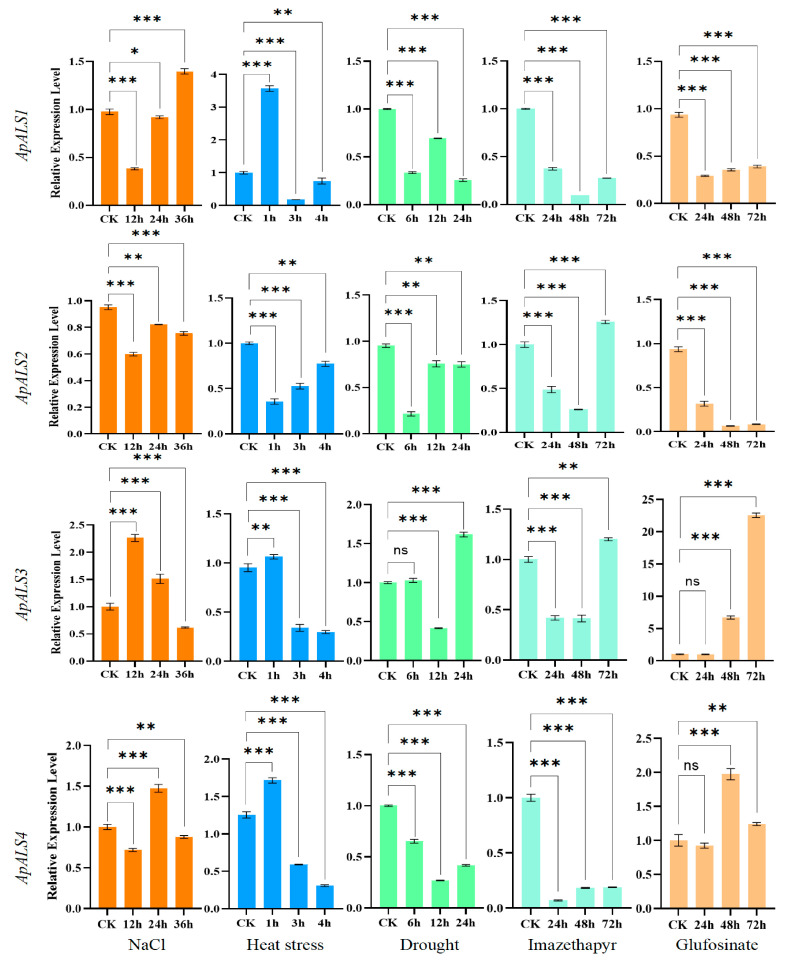
*ApALS*s expression levels under various treatment, including NaCl, heat, osmotic, imazethapyr, and glufosinate ammonium treatments. Significant differences among three biological replicates are indicated by asterisks, reflecting statistical significance at *p* < 0.05. Significance levels are indicated as *p* < 0.0001(***), *p* = 0.0001 (**), *p* < 0.0013 (*), ns—not significant.

To further investigate the expression profiles of *ApALS* genes under stress, RT-qPCR analysis following treatment with NaCl, heat, PEG-6000, glufosinate ammonium, and imazethapyr was performed (Figure 5). After 36 h (h) of salt stress, the expression patterns of *ApALS* genes were highly divergent. The relative expression of *ApALS1* decreased initially but increased thereafter, exceeding control levels at 36 h. *ApALS2* expression levels were downregulated throughout the treatment periods, while *ApALS3* was significantly upregulated at 12 h before gradually declining. *ApALS4* expression upregulated and peaked after 24 h before declining to a downregulation level at 36 h. Under heat stress, *ApALS3* and *ApALS4* expression levels were induced at 1 h but decreased progressively over time. Conversely, *ApALS2* expression remained downregulated throughout the treatment duration; however, the expression levels increased with increasing treatment periods. *ApALS1* expression was initially upregulated and peaked after 1 h before declining compared to the control. Following 24 h of PEG6000-induced osmotic stress, the expression of *ApALS1*, *ApALS2*, and *ApALS4* was lower compared to the control group. In contrast, *ApALS3* expression levels after 12 h treatment were upregulated at the 24 h time point. The two herbicides elicited distinct expression responses. Both glufosinate ammonium and imazethapyr were downregulated in *ApALS1* and *ApALS2*. However, they produced contrasting regulatory effects on the expression of *ApALS3* and *ApALS4*.

### 2.5. Subcellular Localization Analysis of ApALS Genes

**Figure 6:**
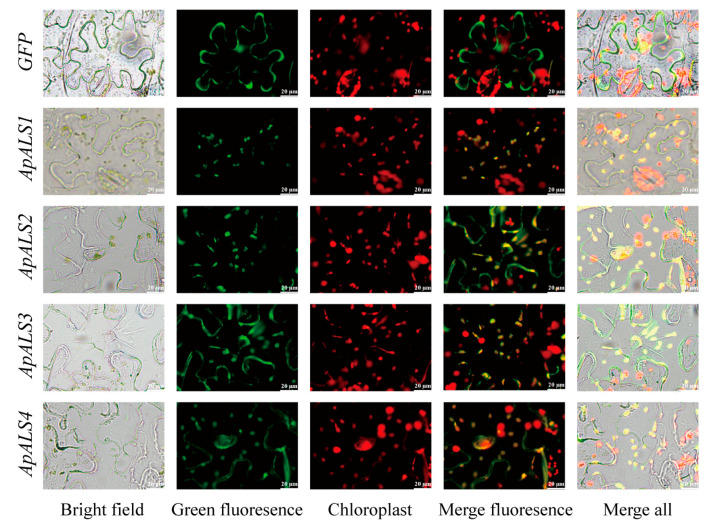
Subcellular localization of ApALSs-GFP fusion protein and empty Pcambia1302-GFP vector through transient expression in *N. benthamiana* leaves.

Subcellular localization predictions indicated that all ApALS proteins are targeted to the chloroplast. To validate these predictions experimentally, a transient expression assay in *Nicotiana benthamiana* leaves was conducted. Expression of fusion proteins consisting of the full length of the ApALSs sequences linked to GFP under the control of the 35S promoter was performed. An empty 35S:GFP vector was used as a control, and, as expected, its fluorescence was distributed throughout the entire cell. In contrast, the fluorescence signals from the ApALSs-GFP fusion proteins were specifically localized to the chloroplasts, which was consistent with the in silico predictions (Figure 6). These results confirm that ApALSs proteins are chloroplast-localized.

### 2.6. Role of the AtALS1 in Imazethapyr Resistance

**Figure 7:**
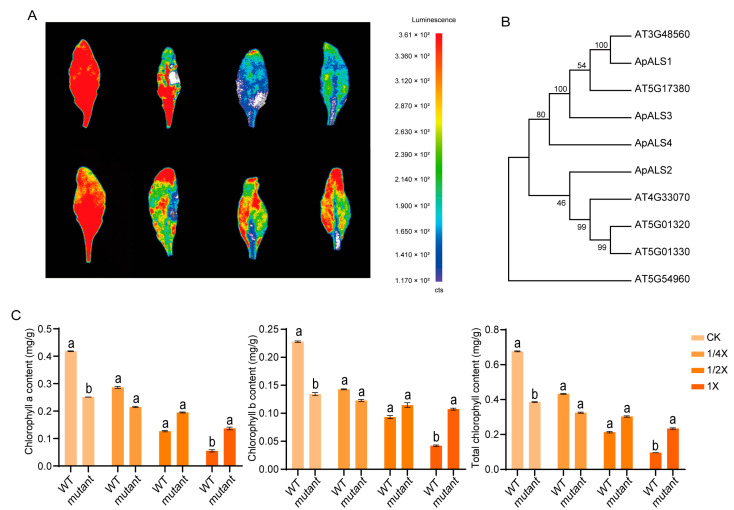
Chlorophyll fluorescence, chlorophyll content of mutant and wild-type Arabidopsis, and phylogenetic tree of ApALS and AtALS proteins. Chlorophyll fluorescence of imazethapyr treated *AT3G48560*-mutant and wild-type Arabidopsis (**A**). Chlorophyll content of imazethapyr treated *AT3G48560*-mutant and wild-type Arabidopsis. Significance levels are indicated by lower-case letters suggesting significant difference (*p* < 0.05) among the mean values (**B**). Phylogenetic tree of ApALS and AtALS proteins (**C**).

Imazethapyr treatment induced chlorophyll degradation in both wild-type (WT) and *AT3G48560*-knockdown mutant *A. thaliana* plants, resulting in visible leaf yellowing (Figure 7A). However, chlorophyll fluorescence intensity, indicative of photosynthetic capacity, remained significantly higher in the mutant plants than in WT plants at all herbicide concentrations tested. Notably, under non-stress conditions, WT plants exhibited higher levels of chlorophyll a, chlorophyll b, and total chlorophyll than the mutant (Figure 7B). In contrast, under imazethapyr stress, this relationship was reversed: the mutant plants retained higher concentrations of all chlorophyll components compared to the stressed WT plants.

Sequence homology between ApALS and AtALS proteins was analyzed using the ClustalW alignment algorithm and the gene duplication function in MEGA 11 software with default parameters. Phylogenetic analysis clustered the ApALS and AtALS sequences into two distinct subgroups. Notably, AT3G48560 and ApALS1 co-clustered on a single terminal branch (Figure 7C), indicating a high degree of sequence homology between them. This close evolutionary relationship suggests that *ApALS1* and *AT3G48560* may perform parallel functional roles in conferring herbicide tolerance, implying that the induction of *ApALS1* expression under stress conditions in *A. palmeri* likely represents a key adaptive strategy that may enhance stress resilience by maintaining chlorophyll integrity and preserving photosynthetic function in *A. palmeri*.

## 3. Discussion

ALS enzymes catalyze the first step in the biosynthesis of branched-chain amino acids and serve as a well-established target site for multiple herbicides. Despite extensive characterization of ALS-mediated herbicide resistance, their potential regulatory functions in the stress responses of *A. palmeri* remain largely unexplored. In this study, four *ApALS* genes in *A. palmeri*, which encode proteins with neutral isoelectric points and hydrophilic properties were identified. These physicochemical characteristics are consistent with an enzymatic role in aqueous compartments such as the cytosol or chloroplasts. As a protein’s function is intrinsically linked to its subcellular localization [23], the site of *ApALS* activity was investigated. The findings align with prior research demonstrating the chloroplast localization of ALS proteins in other species, such as *BnALS3* in *Arabidopsis* protoplasts [24] and *NtALS1* in *N. benthamiana* leaves [25]. Similarly, all the ApALS proteins are localized to the chloroplast. This conserved localization across plant species shows the fundamental role of ALS in the chloroplast-based biosynthesis of branched-chain amino acids. Furthermore, this consistent subcellular targeting confirms the chloroplast as the primary site of action for ALS-inhibiting herbicides, providing a rationale for their efficacy and informing the development of novel compounds that target this accessible pathway [26]. Gene expression is regulated after transcription through processes that influence mRNA stability and translation, often mediated by interactions between regulatory factors and UTRs [27]. Analysis of the *ApALS* genes revealed a complete absence of UTRs. This structural feature suggests that for *ApALS*s, post-transcriptional regulation may be predominantly exercised by miRNA targeting within the CDS or intronic region [28]. A study in *Nymphaea colorata* found that a transcription factor gene lacking UTRs (*NcbHLH52*) was targeted by a notably high number of miRNAs (7), compared to 1–4 miRNAs for genes possessing UTRs [29].

*Cis*-acting regulatory elements within gene promoter regions are critical for modulating gene expression in response to diverse environmental and internal stimuli [30]. Consequently, the identification of these promoter elements can provide vital insights into the potential biological functions of their corresponding genes. In this study, the promoter region of *ApALS1* was found to contain numerous light-responsive elements, including AE-box, TCT-motif, GT1-motif, GA-motif, ATCT-motif, and G-box, as well as several elements associated with plant growth and development, phytohormone signaling, and abiotic stress responses. The notable enrichment of these *cis*-elements in the *ApALS1* promoter may be attributed to its relatively simple gene structure, which lacks complex intronic processing. This architectural feature could allow for the rapid induction of *ApALS1* expression directly through promoter activation upon environmental perception, facilitating a swift metabolic adjustment to fluctuating light and stress conditions [31]. These findings suggest that *ApALS1* likely plays a potential role in coordinating photosynthesis and stress response pathways [32].

Imazethapyr belongs to the imidazolinone class of Group 2 herbicides and is widely utilized for its broad-spectrum efficacy against grasses, sedges, and broadleaf weeds. Imazethapyr is widely applied to control broadleaf weeds in major crops such as soybean, wheat, corn, sorghum, and olive trees globally [33,34,35]. In this study, gene expression analysis revealed that *ApALS2* and *ApALS3* were upregulated at 48 h and 72 h following imazethapyr treatment, suggesting their involvement in the late-phase stress response of *A. palmeri*. This upregulation may represent a compensatory mechanism aimed at restoring branched-chain amino acid biosynthesis under herbicide-induced pressure [36]. In contrast, *ApALS1* and *ApALS4* were downregulated, indicating that these isoforms may either be negatively regulated or not directly involved in the imazethapyr-triggered stress response. Known resistance-conferring mutations in the *ALS* gene, including *Pro197Ser* and *Trp574Leu*, are reported to diminish the binding affinity of imazethapyr [37,38]. In rice, the *Pro171Phe* ALS mutation conferred resistance to bispyribac-sodium [39]. A previous study demonstrated that the ALS herbicide inhibitor can be transferred and expressed from *A. palmeri* to *A. spinosus*, resulting in cross-resistance to multiple herbicides, including imazethapyr [40].

In the present study, *ApALS3* and *ApALS4* were upregulated following glufosinate ammonium treatment at 48 and 72 h, whereas *ApALS1* and *ApALS2* were consistently downregulated throughout the treatment period. These results suggest that *ApALS3* and *ApALS4* may play a role in the stress-responsive regulation of ALS-related pathways under glufosinate exposure and contribute to metabolic detoxification. Conversely, the downregulation of ApALS1 and ApALS2 under glufosinate treatment likely reflects a general stress-induced transcriptional response to ROS accumulation [41]. Additionally, both early- and late-phase transcriptional responses of *ApALS* genes to NaCl, high temperature, and osmotic stress treatments were found, indicating that *ApALS* genes may participate in broader abiotic stress response pathways in *A. palmeri*. This suggests a potential regulatory role for *ApALS* genes contributing to metabolic adaptation under adverse environmental conditions beyond herbicide resistance. The induction of target gene expression under phytotoxic stress may serve as a protective compensatory mechanism to sustain metabolic function and mitigate herbicide damage [35,42]. *Brassica napus*, both natural and CRISPR/Cas9-induced mutations in ALS have produced diverse germplasm with varying levels of resistance to ALS-inhibiting herbicides. These studies indicate that sequence variation in *ALS* genes, and their corresponding amino acid substitutions, can result in a spectrum of herbicide resistance phenotypes within a single species [43,44].

The results demonstrate the key role of the ALS gene in mediating herbicide resistance and maintaining photosynthetic function under imazethapyr-induced stress. In *A. thaliana*, exposure to imazethapyr led to chlorophyll degradation in both WT and *AT3G48560*-knockdown mutant lines. However, chlorophyll fluorescence remained consistently higher in the mutant across all herbicide concentrations, indicating enhanced tolerance. Under non-stress conditions, WT plants exhibited higher levels of chlorophyll a, chlorophyll b, and total chlorophyll. This pattern reversed under herbicide treatment, with mutant plants retaining significantly more chlorophyll, suggesting a protective mechanism linked to ALS gene suppression. Downregulation or mutation of ALS can confer resistance to imazethapyr, potentially by sustaining chlorophyll content and preserving photosynthetic capacity [10,14]. Although ALS is essential for branched-chain amino acid biosynthesis, reduced expression may activate compensatory stress pathways or modify herbicide uptake and metabolism, potentially contributing to imazethapyr tolerance. Comparable non-canonical resistance mechanisms have been documented enhanced detoxification. Therefore, the observed phenotype may result from off-target physiological adaptations rather than direct ALS-mediated resistance [45]. Phylogenetic analysis revealed that *ApALS1* co-clusters with *AT3G48560* on a terminal branch, indicating strong sequence homology and suggesting functional conservation between the two genes. This evolutionary relationship implies that *ApALS1* may perform a comparable role in *A. palmeri* and mitigate herbicide-induced stress through mechanisms that stabilize chlorophyll levels and maintain photosynthetic efficiency. Given that *ApALS1* is localized to the chloroplast, it may be involved in chlorophyll preservation and photosynthetic resilience under stress conditions in *A. palmeri* [26]. Further functional characterization of *ApALS* genes is necessary to investigate their specific contributions to the robust stress adaptation strategies observed in *A. palmeri*.

The results of this study provide valuable insights into herbicide resistance mechanisms and their potential application in sustainable weed management. The observed stress response under various treatments, including imazethapyr and glufosinate-ammonium, implies a potential regulatory role of *ApALS* genes in herbicide sensitivity and metabolic adaptation, consistent with previous findings on ALS-mediated resistance pathways. These findings support the use of ALS-targeted screening for resistant biotypes and inform strategies such as herbicide rotation, dose optimization, and integrated weed management to delay resistance evolution [46]. Moreover, the molecular characterization of ALS variants and promoter architecture may aid in developing predictive tools for resistance monitoring and precision agriculture [47]. As herbicide resistance continues to challenge global food security, integrating genetic insights with field-level practices is essential for long-term sustainability.

## 4. Materials and Methods

### 4.1. Identification and Sequence Analysis of ApALS Proteins

The *ApALS* gene family was identified using a dual approach. First, the annotated genome of *A. palmeri* was acquired from the Comparative Genomics (CoGe) database (https://genomevolution.org/coge/ (accessed on 15 November 2024)). A Hidden Markov Model (HMM) profile for the ALS domain (PF00205) was retrieved from the Pfam database (http://pfam-legacy.xfam.org/ (accessed on 15 November 2024)). The Simple HMM Search function in TBtools v2.310 [25] was then employed to screen the *A. palmeri* proteome against this HMM profile to identify potential ALS sequences. Second, known ALS protein sequences from *A. thaliana* [48], *Zea mays* [49], *Oryza sativa* [50], and *Cyperus difformis* [51] were obtained from the NCBI (https://www.ncbi.nlm.nih.gov/ (accessed on 19 November 2024)) and Ensembl Plants (https://plants.ensembl.org/ (accessed on 19 November 2024)) databases. A local BLASTp search was conducted using these sequences as queries against the *A. palmeri* proteome with the Blast Several Sequences to a Big Database tool in TBtools, applying an E-value cut-off of 1e-5. The candidate ApALSs proteins identified through these two methods were merged and screened using the NCBI conserved domain (CDDv3.21-62456 PSSMs), SMART (http://smart.embl.de/ (accessed on 21 November 2024)), and Pfam [52] databases, using default settings to eliminate unmatched, redundant, and incomplete protein sequences.

### 4.2. Physicochemical Properties of ApALS Proteins

The physicochemical properties of the identified ApALS proteins, including amino acid length (aa), molecular weight (MW), theoretical isoelectric point (pI), grand average of hydropathicity (GRAVY), and instability index, were predicted using the ProtParam tool on the ExPASy server (https://web.expasy.org/protparam/ (accessed on 22 November 2024)). The presence of a signal peptide was assessed with SignalP 6.0 (https://services.healthtech.dtu.dk/services/SignalP-6.0/ (accessed on 22 November 2024)), while chloroplast transit peptide (cTP) was predicted by TargetP-2.0 (https://services.healthtech.dtu.dk/services/TargetP-2.0/ (1 October 2025)). Subcellular localization predictions were performed using Plant-mPLoc (http://www.csbio.sjtu.edu.cn/bioinf/plant-multi/ (accessed on 22 November 2024)). Furthermore, the three-dimensional structures of the ApALS proteins were modeled using the SWISS-MODEL homology modeling server (https://swissmodel.expasy.org/ (accessed on 22 November 2024)).

### 4.3. Chromosomal Distribution of ApALSs

Genomic annotation data for the identified *ApALS* genes were extracted from the *A. palmeri* genome annotation file (GFF). The genomic locations of these genes, including their start and end coordinates, were mapped to their respective scaffolds and visualized using the Gene Location Visualizer tool in TBtools.

### 4.4. Phylogenetic Analysis of ApALSs

ALS protein sequences from *Amaranthus cruentus*, *A. tuberculatus*, *A. hybridus*, *A. hypochondriacus*, *A. thaliana*, *Oryza sativa*, *Portulaca amilis*, and *Zea mays* were retrieved from the NCBI, Phytozome v13, and Amaranth Genomic Resource (AGRDB) databases [53] via a BLASTp search with an E-value cutoff of 1 × 10^−5^. The retrieved protein sequences were screened using the NCBI-CDD and SMART databases to confirm the presence of the three ALS characteristic domains: TPP_enzyme_N, TPP_enzyme_M, and TPP_enzyme_C. A phylogenetic tree was constructed to evaluate the evolutionary relationships among 52 ALS proteins, including the ApALSs, AcALSs, AtuALSs, AhALSs, AhyALSs, AtALSs, OsALSs, PaALSs, and ZmALSs. Multiple sequence alignment was performed using ClustalW with default parameters. The phylogenetic tree was then constructed using the Neighbor-Joining (NJ) method. The robustness of the tree topology was assessed by a bootstrap analysis with 1000 replicates, applying the Jones–Taylor–Thornton (JTT) model, uniform rates, and pairwise deletion [54]. Finally, the tree was visualized using the iTOL platform [55].

### 4.5. Gene Structure, Motif, Cis-Acting Element, and miRNA-ApALSs Targets Analyses

The exon–intron structures of the *ApALS* genes were determined based on the *A. palmeri* genome annotation and visualized using the Gene Structure View function in TBtools. To characterize the conserved motifs within the ApALS protein sequences, analysis was conducted using the MEME suite [56]. Furthermore, a 2.0 kb genomic sequence upstream of each *ApALS* transcription start site was analyzed with the PlantCARE database to predict putative *cis*-acting regulatory elements [57]. The resulting data from the gene structure, protein motif, and promoter element analyses were integrated and visualized using TBtools. Mature miRNAs of *A. palmeri* were retrieved from the AGRDB database. To predict miRNA targets, the CDS of the *ApALS* genes were analyzed against these miRNAs using the psRNATarget online platform with default parameters [58]. The resulting interaction network was visualized using Cytoscape v3.10.2.

### 4.6. Plant Materials and Growth Conditions

A herbicide-resistant *A. palmeri* population was collected from Jingzhou, Hubei Province, where repeated field applications of ALS- and EPSPS-inhibiting herbicides had failed to control infestations. Mature plants that survived these treatments were harvested for further analysis. Plants were subsequently cultivated in a controlled glasshouse environment under the following conditions: a 16/8 h light/dark photoperiod with temperatures of 30 °C (light) and 28 °C (dark), and 55% relative humidity. Both WT *A. thaliana* and the *ALS* T-DNA insertion mutant line *WiscDsLox435E9* (*At3g48560*) were grown concurrently in the same greenhouse.

### 4.7. Stress Treatment Conditions of A. palmeri

The seeds were surface-sterilized using a 1% hypochlorite solution, thoroughly washed with distilled water, and germinated on petri dishes with filter paper. The seedlings were transferred to a hydroponics tray with a half-strength diluted Hoagland nutrient solution. At the 4–6 leaf stage, treatments were performed with NaCl (200 mM), heat (40 °C), osmotic (20% PEG6000), imazethapyr (0.375 g/L), and glufosinate-ammonium (2.25 g/L). Samples were collected at 0 h, 12 h, 24 h, 36 h, 48 h, and 72 h post-treatment and were immediately frozen in liquid nitrogen and stored at −80 °C for subsequent analysis. Three biological replicates were maintained for each treatment.

### 4.8. Real-Time Quantitative PCR (RT-qPCR) Analysis

Total RNA was extracted using Trizol reagent (Invitrogen, Gaithersburg, MD, USA). Using 1.0% agarose gel, RNA integrity was examined through electrophoresis, and the concentration and purity of the RNA were determined using a NanoDrop 2000 spectrophotometer. RNA samples with RIN values greater than 7 were used for cDNA synthesis using HiScript Reverse Transcriptase (Vazyme, Nanjing, China) according to the manufacturer’s protocol. The RT-qPCR analysis was performed on the MA-6000 Real-Time Quantitative Thermal Cycler using ChamQ SYBR qPCR Master Mix (Vazyme, Nanjing, China), according to the manufacturer’s instructions. Primer Premier 5 was used to design specificity primers, with UBQ as the internal reference gene (Supplementary File S1). The 20 μL reaction system consists of 10 μL 2 × ChamQ SYBR qPCR Master Mix, 0.4 μL each of forward and reverse primers, 1 μL diluted cDNA, and 8.2 μL ddH2O. The RT-qPCR reaction procedure was as follows: pre-denaturation at 95 °C for 3 min (Step 1); 95 °C denaturation for 10 s (Step 2); annealing at 55 °C for 30 s (Step 3) and at 65 °C for 5 s (Step 4); 40 cycles from Step 2 to 3. Three technical replicates were performed for each cDNA, and the relative expression of *ApALSs* was calculated by the 2^−∆∆CT^ method [59]. A two-way ANOVA was performed to evaluate the statistical differences of the *ApALSs* expression levels between the treatment and control groups using GraphPad Prism v10.2.0 with a *p*-value of ≤0.05 being statistically significant [60].

### 4.9. Subcellular Localization Analysis of ApALSs

The subcellular localization of *ApALSs* was predicted using the online software WoLF PSORT (https://wolfpsort.hgc.jp/ (accessed on 30 November 2024)). To confirm the predicted location of *ApALSs*, the full-length coding sequence (CDS) of the *ApALS1* and *ApALS4* were amplified by PCR (Supplementary File S1) and fused to the C-terminus of the green fluorescent protein (GFP) gene in the pCAMBIA1302-GFP overexpression vector. The recombinant plasmid *ApALS*-GFP was inserted into Agrobacterium GV3101 using the freeze-thaw method. The transformed GV3101 was cultured in solid LB medium at 28 °C for 48 h. A single positive colony was then inoculated into fresh medium and shaken overnight. The bacterial cells were centrifuged and resuspended in infiltration buffer. For transient expression, the *Agrobacterium* suspension was injected into the abaxial side of 4-week-old *N. benthamiana* leaves using a needleless syringe. The empty pCAMBIA1302-GFP vector served as a positive control for GFP localization. After infiltration, the plants were kept in the dark for 24 h and then grown under normal light conditions for 48 h before imaging. GFP fluorescence was observed using a laser scanning confocal microscope (Model BX53F2C, Yijingtong Optical Technology Co., Ltd., Shanghai, China) under 488 nm excitation and 510 nm emission.

### 4.10. Herbicide Resistance Assay in Mutant Plants

To investigate the role of ALS in plant stress resistance, herbicide stress experiments were conducted using the *A. thaliana* T-DNA insertion knockdown mutant *WiscDsLox435E9* (*AT3G48560*), alongside the WT. The mutant line was obtained from the AraShare Technology Service Center. Seeds were surface-sterilized with 75% (*v*/*v*) ethanol and stratified at 4 °C in the dark for three days to synchronize germination. They were then germinated on solid Murashige and Skoog (MS) medium supplemented with 2% (*w*/*v*) sucrose and 0.8% (*w*/*v*) agar [61]. After two weeks, seedlings were transplanted into a 1:3 (*v*/*v*) mixture of peat moss and vermiculite and maintained under identical growth conditions. Confirmed homozygous progeny were used for subsequent experiments. Herbicide treatments were applied at the 4–6 leaf stage using a hand-held sprayer calibrated to deliver a uniform spray volume of 200 L ha^−1^. Plants were treated with imazethapyr at three concentrations: 0.375 g/L (1× field-recommended rate), 0.1875 g/L (1/2×), and 0.09375 g/L (1/4×), as previously described [62]. Control plants were sprayed with distilled water. Each treatment group consisted of three biological replicates, and leaves were harvested for analysis three days post-treatment. The entire experiment was independently replicated at least three times.

Chlorophyll fluorescence was visualized to assess photosynthetic performance. Leaves from treated seedlings were immediately imaged using a charge-coupled device (CCD) camera in a PlantView 100 system (Guangzhou Boluteng Biotechnology Co., Ltd., China) under the following settings: light-emitting camera mode, 60-s exposure time, and a binning value of 2 × 2 [63]. Chlorophyll content was quantified spectrophotometrically. Fresh leaf tissue was homogenized, and chlorophyll was extracted. A 200 µL aliquot of the supernatant was transferred to a 96-well plate, with 200 µL of extraction buffer serving as the blank. Absorbance was measured at 665 nm and 649 nm using a microplate reader. Chlorophyll a, b, and total concentrations were calculated according to the following equations:

Chlorophyll a (mg/L) = (13.95 × A_665_) − (6.88 × A_649_)

Chlorophyll b (mg/L) = (24.96 × A_649_) − (7.32 × A_665_)

Total Chlorophyll (mg/L) = (6.63 × A_665_) + (18.08 × A_649_)

### 4.11. Statistical Analysis

To evaluate statistical differences between the two experimental groups, a Student’s *t*-test was applied. The data were derived from three independent biological replicates and were expressed as the mean ± standard error of the mean (SEM). Graphs were generated using GraphPad Prism software v9.5.

## 5. Conclusions

This study characterized the ALS gene family in *A. palmeri* and investigated its role in mediating responses to diverse stress conditions. The study identified four *ApALS* genes, which potentially play a key function in chloroplast-localized biosynthetic processes during *A. palmeri* stress conditions. Non-canonical miRNA targeting within coding sequences and introns was revealed as a possible post-transcriptional regulatory mechanism influencing *ApALS* gene expression. Expression profiling indicated that *ApALS* genes are responsive to multiple stress treatments, including salinity, heat, osmotic, imazethapyr, and glufosinate ammonium. Functional analysis using an *A*. *thaliana* ALS T-DNA mutant demonstrated alterations in chlorophyll content and photosynthetic activity under imazethapyr exposure. Homology-based comparisons further suggested that *ApALS1* may play a significant role in regulating chlorophyll metabolism and photosynthesis in *A. palmeri* during herbicide stress. Further research is required to investigate the regulatory mechanisms governing *ApALS* gene expression under diverse abiotic and herbicide-induced stress conditions. This study provides key insights into the evolution and function of the ALS gene family in a major weed species and establishes a foundation for future research aimed at exploiting these genes. Such research could lead to novel strategies for controlling *A. palmeri* infestations and provide valuable genetic targets for enhancing stress tolerance in crop plants through molecular breeding.

## Data Availability

The data presented in this study are available on request from the corresponding author.

## Supplementary Materials

The following supporting information can be downloaded at: https://www.mdpi.com/article/10.3390/plants14193088/s1, Supplementary File S1. Primers used in this study; Supplementary File S2. Physicochemical properties of the ApALS proteins; Supplementary File S3. Protein secondary structure of ApALS proteins; Supplementary File S4. Conserved motifs of the ApALS proteins; Supplementary File S5. MiRNA-ApALS Target Interaction.
